# Dual targeting of glutamine and serine metabolism in acute myeloid leukemia

**DOI:** 10.3389/fonc.2024.1326754

**Published:** 2024-04-16

**Authors:** Kanwal M. Hameed, Dominique R. Bollino, Amol C. Shetty, Brandon Carter-Cooper, Rena G. Lapidus, Ashkan Emadi

**Affiliations:** ^1^ School of Medicine, University of Maryland, Baltimore, Baltimore, MD, United States; ^2^ University of Maryland Marlene and Stewart Greenebaum Comprehensive Cancer Center, Baltimore, MD, United States; ^3^ Department of Medicine, School of Medicine, University of Maryland, Baltimore, Baltimore, MD, United States; ^4^ Institute of Genome Sciences, School of Medicine, University of Maryland, Baltimore, Baltimore, MD, United States; ^5^ Department of Pharmacology, School of Medicine, University of Maryland, Baltimore, Baltimore, MD, United States

**Keywords:** cancer metabolism, asparaginase, glutamine, serine, leukemia

## Abstract

Acute myeloid leukemia (AML) is a heterogeneous hematological malignancy characterized by disrupted blood cell production and function. Recent investigations have highlighted the potential of targeting glutamine metabolism as a promising therapeutic approach for AML. Asparaginases, enzymes that deplete circulating glutamine and asparagine, are approved for the treatment of acute lymphoblastic leukemia, but are also under investigation in AML, with promising results. We previously reported an elevation in plasma serine levels following treatment with *Erwinia*-derived asparaginase (also called crisantaspase). This led us to hypothesize that AML cells initiate the *de novo* serine biosynthesis pathway in response to crisantaspase treatment and that inhibiting this pathway in combination with crisantaspase would enhance AML cell death. Here we report that in AML cell lines, treatment with the clinically available crisantaspase, Rylaze, upregulates the serine biosynthesis enzymes phosphoglycerate dehydrogenase (PHGDH) and phosphoserine aminotransferase (PSAT1) through activation of the Amino Acid Response (AAR) pathway, a cellular stress response mechanism that regulates amino acid metabolism and protein synthesis under conditions of nutrient limitation. Inhibition of serine biosynthesis through CRISPR-*Cas9*-mediated knockout of PHGDH resulted in a ~250-fold reduction in the half-maximal inhibitory concentration (IC_50_) for Rylaze, indicating heightened sensitivity to crisantaspase therapy. Treatment of AML cells with a combination of Rylaze and a small molecule inhibitor of PHGDH (BI4916) revealed synergistic anti-proliferative effects in both cell lines and primary AML patient samples. Rylaze-BI4916 treatment in AML cell lines led to the inhibition of cap-dependent mRNA translation and protein synthesis, as well as a marked decrease in intracellular glutathione levels, a critical cellular antioxidant. Collectively, our results highlight the clinical potential of targeting serine biosynthesis in combination with crisantaspase as a novel therapeutic strategy for AML.

## 1 Introduction

Acute myeloid leukemia (AML) is an aggressive and heterogeneous hematological malignancy characterized by the clonal expansion of abnormal myeloid progenitors in the bone marrow. Despite recent approvals for new therapeutic agents, the 5-year survival remains poor (1, 2). As ongoing research continues to unravel the complex biology of AML, the identification of novel therapeutic targets and the development of innovative treatment regimens holds great promise for improving patient outcomes. AML development is influenced by metabolic reprogramming, which introduces vulnerabilities that can be targeted for cancer treatment (3). For example, AML cells are not able to produce enough glutamine to meet cellular energy demand, rendering them reliant on circulating levels (4). Targeting glutamine has therefore emerged as a novel therapeutic strategy for AML (5–7).

L-asparaginases (L-ASNase) are enzymes that facilitate the hydrolysis of asparagine into aspartate and glutamine into glutamate, thereby reducing circulating levels of these amino acids. Clinically available L-ASNases are derived from two bacterial sources, *Escherichia coli* (*E*. *coli*), and *Erwinia chrysanthemi*, (also called crisantaspase). Crisantaspase possesses a unique advantage in its higher glutaminase activity compared to *E. coli*-derived L-ASNase (8, 9). L-ASNase is currently FDA-approved for the treatment of acute lymphoblastic leukemia (ALL) and lymphoblastic lymphoma; however, prior research from our lab and others has demonstrated that L-ASNase is also effective against AML (6, 7, 10–12).

We recently reported that crisantaspase treatment led to elevated plasma levels of serine in murine models as well as in human AML samples from a phase 1 clinical trial (5). Serine is a versatile amino acid with roles in nucleotide synthesis, redox homeostasis maintenance, and protein glycosylation (13, 14) and AML cells have been shown to be dependent on serine for cell survival and growth, particularly when glutamine metabolism is disturbed (12). We therefore hypothesized that crisantaspase treatment induces *de novo* serine biosynthesis in response to glutamine depletion and that inhibiting this pathway would enhance crisantaspase-induced AML cell death. To test this hypothesis, we used the FDA-approved crisantaspase, Rylaze, and investigated the mechanism underlying the crisantaspase-mediated upregulation of serine and whether targeting serine metabolism potentiates the effect of Rylaze.

## 2 Materials and methods

### 2.1 Cell lines and culturing

The human AML cell lines MOLM-14, MonoMac-6, MV4-11, U937, HL60, and K562 cells were provided by the Translational Laboratory Shared Service, University of Maryland, Baltimore. Ba/F3 WT and *FLT3*-ITD were a gift from Dr. Feyruz Rassool (University of Maryland, Baltimore). All cell lines were grown at 37°C with a 5% CO_2_ atmosphere in Roswell Park Memorial Institute (RPMI) 1640 medium (Life Technologies, Carlsbad, CA, Cat#:11875119) supplemented with heat-inactivated 10% (V/V) fetal bovine serum (FBS). For the experiments requiring medium adaptation, RPMI 1640 medium without L-glutamine (Life Technologies, Cat#: 21870076) was used and supplemented with L-glutamine (Thermo Fisher Scientific, Waltham, MA, Cat#: A2916801) to achieve the desired concentrations. Cell lines were grown and maintained according to ATCC recommendations. All cells were tested for mycoplasma contamination and utilized before 20 passages and treated in the exponential growth phase at ~70% confluence.

Primary human AML cells were obtained through an IRB-approved institutional tissue procurement protocol at the University of Maryland. Briefly, whole blood and bone marrow aspirates were received in sodium heparin or ethylenediaminetetraacetic acid (EDTA) tubes and diluted 1:1 with phosphate-buffered saline (PBS). Cells were isolated using lymphocyte separation medium (Corning Cellgro, Manassas, VA, Cat#: 25-072-CI) and viable cell numbers were calculated using trypan blue. Primary cells were cultured in X-Vivo 15 Serum-free Hematopoietic cell medium (Lonza, Basel, Switzerland, Cat#: 02-060Q) supplemented with granulocyte-macrophage colony-stimulating factor (GM-CSF) (Cell Signaling Technology, Danvers, MA Cat#: 8930) at 10 ng/mL, interleukin 3 (IL3) (Cell Signaling Technology, Cat#: 8918) at 50 ng/mL, recombinant human thrombopoietin (TPO, Biolegend, San Diego, CA, Cat#: 712602) at 50 ng/mL, stem cell factor (SCF) (Cell Signaling Technology, Cat#: 9907) at 25 ng/mL, FLT3 ligand (FLT3L) (Peprotech, Rocky Hill, NJ, Cat#: 300-19) at 50 ng/mL, interleukin 6 (IL6) (Cell Signaling Technology, Cat#: 9954) at 10 ng/mL, and granulocyte colony-stimulating factor (G-CSF) (Cell Signaling Technology, Cat#: #8930) at 1 ng/mL.

### 2.2 Reagents and chemotherapeutics

Rylaze was purchased from Jazz Pharmaceuticals. BI4916 (Cat#: HY-126253) and GCN2-IN-1 (Cat#: HY-100877) was purchased from MedChem Express (Monmouth Junction, NJ) as a powder and dissolved in DMSO in 50 mM stock solutions and stored at -20°C.

### 2.3 Cell proliferation assay

Cell lines and primary cells were seeded into 96-well plates the afternoon before treatment and approximately 18 hours (h) after plating, Rylaze (0-1 µg/mL) and BI4916 (0-50 µM) were serially diluted in growth medium and added to cells. Cell lines were incubated for 72 h, and primary cells for 48 h before the addition of water-soluble tetrazolium (WST-1) (Takara Bio, Kusatsu, Shiga, Japan, Cat#: MK400) for cell lines and AlamarBlue (BioRad, Hercules, CA, Cat#: BUF012A) for primary cells. After 4 h of additional incubation at 37°C, plates were read using a BioTek Synergy HT plate reader (BioTek, Winooski, VT). Data were analyzed and graphed using GraphPad Prism Software (GraphPad, La Jolla, CA), and IC_50_ concentrations were calculated.

### 2.4 Combination index

For synergism, both BI4916 (0-50 µM) and Rylaze (0-1 µg/mL) were added in fixed ratios simultaneously, and cell line cultures were terminated 72 h after treatment with WST-1 reagent. Primary AML sample cultures were terminated at 48 h with the addition of AlamarBlue. After 4 h of additional incubation, plates were read at 37°C using a BioTek Synergy HT plate reader. Data were analyzed by median effect analysis using the Compusyn Software (free online software based on the Chou Talalay theorem) (15). A combination Index (CI) was generated; CI<1 synergistic, CI=1 additive, CI>1 antagonistic.

### 2.5 Cell cycle analysis

MOLM-14 cells were plated overnight and treated with vehicle or Rylaze (IC_50_ concentration). After 48 h the cells were washed then fixed and permeabilized with cold 70% ethanol for 2 h at -20°C then washed with cold PBS twice. Cells were then resuspended in a staining buffer [PBS with 0.5% BSA, 2 mM EDTA, 100 µg/mL RNAse (Sigma Aldrich, Burlington, MA, Cat#: 10109142001) and propidium iodide (BioLegend, Cat#: 421301)] and incubated for 1 h at 4°C. After staining, samples were run on BD FACS Canto II and analyzed using FCS Express V6 (*De Novo* Software, Pasadena, CA).

### 2.6 Measurement of serine levels

Serine was measured using the DL-Serine Assay Kit (Sigma Aldrich, Burlington, MA, Cat#: MAK352), a fluorescent-based assay for detecting and quantifying serine concentration, according to manufacturer’s instructions. MOLM-14 and U937 cells lines were seeded at 1x10^6^/well in a 6-well plate overnight and treated with vehicle (DMSO) and Rylaze. After 72 h, 10 µL of supernatant was added to a 96-well plate with the addition of the following reaction mixes to detect for: D-Serine only, Total Serine and Sample Background to the respective wells. The plate was incubated at 37°C, protected from light for 60 minutes. Fluorescence was read at Ex/Em = 535/587nm on BioTek HT Synergy Plate Reader. Sample background, of serine content in media was subtracted from experimental values. Data were analyzed using GraphPad Prism.

### 2.7 Measurement of glutathione levels

Glutathione (GSH) was measured using the GSH-Glo Glutathione Assay kit (Promega, Cat#: V6911), a luminescence-based assay for detecting and quantifying GSH. MOLM-14 and U937 cell lines were seeded at 5x10^4^/well in a 96-well plate overnight then treated with vehicle (DMSO), BI4916 (2 µM), Rylaze (0.1 µg/mL) or BI4916-Rylaze combination. After 72 h, the supernatant was removed, and cells were incubated at room temperature with 1X GSH Glo Reagent. After 30 minutes Luciferase Detection Buffer was added to each well. Luminescence was measured using BioTek HT Synergy Plate Reader 15 minutes after. Data were analyzed using GraphPad Prism Software.

### 2.8 Measurement of reactive oxygen species

To measure Reactive oxygen species (ROS) levels, cells were plated and treated similar to the GSH assay, followed by preloading with 2′,7′-dichlorodihydrofluorescein diacetate (H_2_DCFA, Life Technologies) at a final concentration of 2 µM. After a 25-minute incubation at 37°C, treatment with BI4916 (2 µM), Rylaze (0.1 µg/mL), and their combination. Treatments were carried out in phenol red-free DMEM or RPMI with 10% FBS, and incubation at 37°C. ROS measurements were recorded at 0, 1, 2, 4, 6 and 24 h, using a Bio-Tek Synergy H1 plate reader. Data were presented as relative fluorescent units and analyzed using GraphPad Prism Software.

### 2.9 Measurement of glutamate

Glutamate was measured using the Glutamine/Glutamate-Glo Glutathione Assay kit (Promega, Cat#: J8021), a luminescence-based assay for detecting and quantifying glutamate. MOLM-14 and U937 cell lines were seeded at 5x10^4^/well in a 96-well plate overnight then treated with vehicle (DMSO), BI4916 (2 µM), Rylaze (0.1 µg/mL) or BI4916-Rylaze combination. After 72 h, cells were washed and two 25 µL aliquots were transferred to a 96-well white clear bottom plate and 25 µL of glutaminase buffer with glutaminase was added to the controls. Plate was incubated at room temperature (RT) for 30 minutes, after which 50 µL of luciferase detection reagent was added to all samples. Plate was incubated for 1 h at RT, and luminescence was measured using a Bio-Tek Synergy H1 plate reader.

### 2.10 Western blot analysis

Cells were washed with PBS and then lysed with radioimmunoprecipitation assay (RIPA) buffer (Millipore, Burlington, MA, Cat# 89900) supplemented with EDTA-free Protease/Phosphatase Inhibitor Cocktail (Sigma Aldrich, Cat # 11873580001). After vortexing and incubation at 4°C for 10 minutes, lysates were then centrifuged at 17,000g at 4°C for 15 minutes and the supernatant was collected. The protein content of lysates was determined by Bio-Rad Protein Assay Dye (BioRad, Cat#: #5000001), and the lysates were then separated by 4-15% polyacrylamide gel electrophoresis (SDS-PAGE) (BioRad), and electro-transferred onto polyvinylidene difluoride (PVDF) membranes (BioRad, Cat: Cat # 88518). After blocking with 5% non-fat milk (BioRad) or bovine serum albumin (BSA) (Sigma-Aldrich), in tris-buffered saline, 0.1% Tween 20 (TBST), membranes were incubated with primary antibodies at 4°C overnight. PHGDH, GCN2, ph-GCN2, eIF2α, ph-eIF2α, ATF4, p70S6K, ph-p70S6K, eIF4E, ph-eIF4E, 4EBP1, ph-4EBP1, and GAPDH were purchased from Cell Signaling Technologies (Danvers, MA). PSAT was purchased from Thermo Fisher Scientific. Membranes were then incubated with horseradish peroxidase (HRP)-conjugated secondary antibody (Cell Signaling Technologies, Cat # sc-516102 and Cat # sc-2357-CM) for 1 h at RT. Bands were then visualized using the Enhanced Chemiluminescence (ECL) Western Blot Substrate (Thermo Fisher Scientific, Cat#:32106). Densitometric analyses (n=3) were performed with ImageJ Studio and presented as a ratio of target band signal intensity to GAPDH band signal.

### 2.11 Puromycin incorporation assay (SUnSET assay)

The SUnSET (surface sensing of translation) assay was performed per the manufacturer’s recommendations (Kerafast, Boston, MA). Briefly, cells were treated with BI4916, Rylaze, BI4916 and Rylaze, or DMSO-control for 16 h and then were incubated for 20 minutes with 1 µg/mL puromycin. After incubation at 37°C, cells were washed with PBS and then lysed with RIPA buffer. Protein lysates were separated by SDS-PAGE and immunoblotting was performed with an anti-puromycin antibody (Sigma Aldrich, Cat#: MABE343). Densitometric analyses (n=3) were performed with ImageJ Studio and presented as a ratio of target band signal intensity to GAPDH band signal.

### 2.12 CRISPR-*Cas9*

For CRISPR-*Cas9*-mediated PHGDH knockout in MOLM-14 and U937 cells, single-guide RNAs (sgRNAs) were designed targeting both exon 6 and exon 2 of the PHGDH gene. Nucleofections were performed using the Lonza Amaxa 4D X Unit with an SF buffer system. Nucleofected cells were harvested, and subjected to PCR and Sanger sequencing, and sequences were analyzed using the Interference of CRISPR Edits (ICE) analysis software from Synthego, CA. Cell populations with confirmed edits were plated for single-cell colony expansion, and clones were validated by PCR, sequencing, and ICE analysis.

### 2.13 Analysis of m^7^GTP-sepharose-bound proteins

The affinity purification of proteins associated with the m^7^GTP Sepharose (Jena Biosciences, S Jena, Germany, Cat#: AC-155S) was performed as previously described (7). Briefly, cell lysates prepared after 24 h of treatment with vehicle (DMSO), BI4916 (2 µM), Rylaze (0.1 µg/mL) and their combination were incubated with m^7^GTP‐sepharose beads for 2 h in cap‐binding buffer (40 mM 4-(2-hydroxyethyl)-1-piperazineethanesulfonic acid (HEPES), pH 7.6; 120 mM NaCl; 1 mM EDTA; 0.3% 3-[(3-cholamidopropyl)dimethylammonio]-1-propanesulfonate [CHAPS]). The beads were washed at RT three times in cap‐binding buffer and boiled with 2X loading dye, followed by separation by SDS-PAGE and immunoblotting. Densitometric analyses (n=3) were performed with ImageJ Studio and presented as a ratio of target band signal intensity to GAPDH band signal.

### 2.14 Reverse transcription polymerase chain reaction

MOLM-14 cells were treated with vehicle or Rylaze (IC_50_) for 16 h. Total RNA was extracted using the Nucleospin RNA Plus Kit (Takara Bio, Kusatsu, Shiga, Japan, Cat#: 740984.50) according to the manufacturer’s protocol. Total RNA was reverse transcribed into cDNA using the High-Capacity cDNA Reverse Transcription Kit (Thermo Fisher Scientific Cat # 4368814), followed by quantitative polymerase chain reaction (qPCR) with Power SYBR green PCR master mix (Thermo Fisher Scientific, Cat # 4367659) on the CFX96 Real-Time PCR System (BioRad). The primers were purchased from Integrated DNA Technologies (Coralville, IA). Primer sequences are available upon request.

### 2.15 Transcriptome bioinformatic analyses

The transcriptome samples were sequenced at the Institute for Genome Sciences (IGS), Baltimore, MD using the Illumina HiSeq sequencing platform. Raw sequencing reads generated for each sample were analyzed using the CAVERN transcriptomics analysis pipeline (16). Read quality was assessed using the FastQC toolkit (17) to ensure quality reads for downstream analyses. Reads were aligned to the Human reference genome GRCh38 (available from Ensembl repository) using HISAT2, a fast splice-aware aligner for mapping next-generation sequencing reads (18). Reads were aligned using default parameters to generate the alignment BAM files. Read alignments were assessed to compute gene expression counts for each gene using the HTSeq count tool (19) and the Human reference annotation (GRCh38). The raw read counts were normalized for library size and dispersion of gene expression. The normalized counts were utilized to assess differential gene expression across time using the R package ‘DESeq2’ (20). P-values were generated using a Wald test implemented in DESeq2 and then corrected for multiple hypothesis testing using the Benjamini-Hochberg correction method. Significant DEGs between conditions were determined using a false discovery rate (FDR) of 5% and a minimum log_2_ (fold-change) of ±1. The filtered set of genes were further utilized to assess the enrichment of gene ontology (GO) terms.

### 2.16 Statistical analysis

For mechanistic studies that needed comparisons of the mean across multiple treatment groups, we used the analysis of variance (ANOVA) followed by Bonferroni’s *post hoc* correction. The statistical parameters for each experiment including sample size and statistical significance are reported in the figures/figure legends. Statistical analyses were performed using GraphPad Prism Version 10.0.1 (La Jolla, CA).

## 3 Results

### 3.1 Rylaze inhibits leukemia cell proliferation and expansion

To investigate the impact of Rylaze on cell growth, we treated six human AML cell lines that harbor different cytogenetic and mutational burdens (MOLM-14, MV4-11, U937, HL60, K562, and Monomac-6) (6, 7) and two murine lines (Ba/F3 WT and *FLT3*-ITD) with increasing doses of Rylaze. Treatment with Rylaze resulted in a dose-dependent reduction in AML proliferation, with IC_50_s ranging from 0.3 to 6 nanogram(ng)/mL, demonstrating sensitivity and potent single-agent activity in all AML cell lines (**Supplementary Figure S1A**). Taken into account the pan-sensitivity of all AML cell lines as well as the prevalence of *FLT3* and *DNMT3A* mutations in AML, we selected MOLM-14 (*FLT3*-ITD mutated) and U937 (*DNMT3A* mutated) human AML cell lines to be used in further studies due to their representative profiles.

A nadir plasma asparaginase activity of 0.1 IU/mL, which is comparable to 0.1 µg/mL of Rylaze, has been accepted as a clinically effective and pharmacologically achievable threshold in many treatment protocols, clinical research and by regulatory agencies (21–26). This indicates that concentration of 0.1 µg/mL of Rylaze would provide a reasonable surrogate for clinically relevant plasma asparaginase activity in patients with acute leukemia.

Seeing the substantial impact on AML cell proliferation by Rylaze, we wanted to further investigate its effect on cell growth. MOLM-14 and U937 cell lines were treated with vehicle (DMSO) or Rylaze (0.1 µg/mL) then cell expansion was measured using trypan blue exclusion at 24-, 48-, and 72-hours post treatment. We observed that Rylaze significantly inhibited cell growth compared to the control, as indicated by reduced fold-expansion of cell cultures at 72 h (**Supplementary Figure S1B**). Using the same treatment conditions, we also measured the percentage of dead cells by trypan blue and found that Rylaze significantly reduced the percentage of viable U937 cells at all time points tested and reduced MOLM-14 viability at 72 h post-treatment. This reduction in viability was modest and did not exceed 20% of the cell population for MOLM-14 or 50% for U937 (**Supplementary Figure S1C**). To determine if Rylaze impacts cell cycle progression, MOLM-14 cells were treated with vehicle or Rylaze (IC_50_ dose to ensure cell viability) for 48 h, and propidium iodide was used to label total DNA content. Compared to vehicle-treated cells, Rylaze treatment resulted in a 2-fold decrease in the percentage of cells in the S phase and a 3-fold decrease in the G2 phase, while the percentage of cells in the G1 phase increased 2-fold, indicating a stall in cell cycle progression (**Supplementary Figure S1D**). Taken together, these results suggest that Rylaze inhibits cell growth primarily through cytostatic effects. To confirm that Rylaze-mediated glutamine depletion contributes to the observed inhibition of AML cell growth, we cultured AML cells lines in media with decreasing concentrations of glutamine and assessed cell growth compared to cells cultured in normal growth media. Indeed, the removal of glutamine from the media significantly decreased AML cell growth in a concentration-dependent manner (**Supplementary Figure S1E**).

### 3.2 Transcriptomic analysis of Rylaze in AML cells

To further characterize the impact of Rylaze on AML cellular functions, we performed whole-transcriptome analysis/gene expression profiling (GEP) on MOLM-14 cells treated with either the vehicle control or Rylaze (IC_50_) for 16 h using RNA sequencing (RNA-seq) (**Supplementary Figure S2A, S2B, Supplementary Table S1**). Principal Component Analysis (PCA) of the data revealed distinct clusters for replicates within each treatment group and diverse clusters between the vehicle control and Rylaze-treated groups (**Supplementary Figure S2C**).

**Figure 1:**
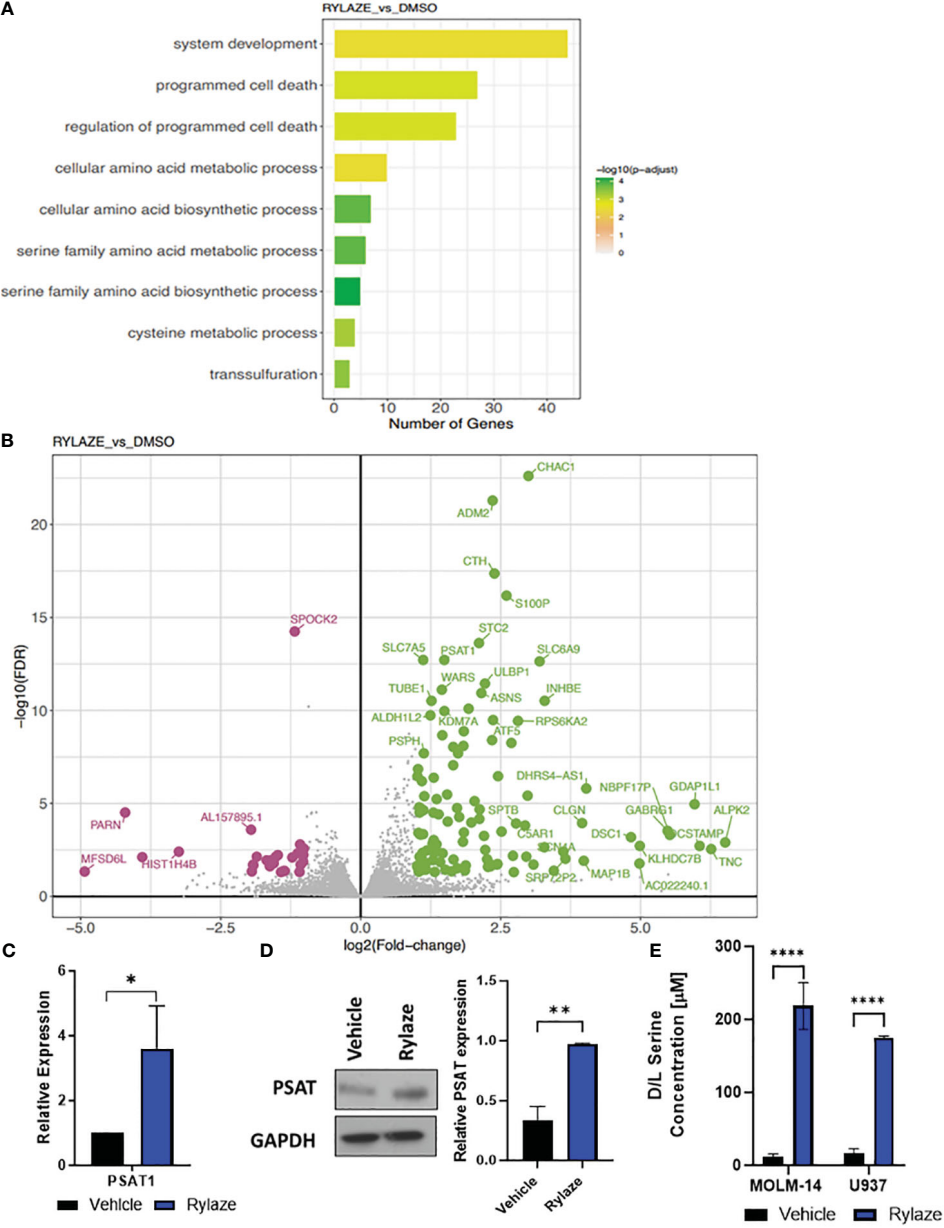
High throughput analysis of Rylaze treatment on MOLM-14 cell lines. **(A)** Bar plot representing the functional Gene Ontology (GO) terms enriched by the differentially expressed genes identified for Rylaze treatment and colored according to the negative log_10_-transformed adjusted p-value. **(B)** Significant up- and down- regulated genes identified between Rylaze treatment and control are shown in green and pink respectively. Differentially expressed genes (DEGs) illustrated with the log_2_(fold-change) (x-axis) and –log_10_ transformed adjusted p-value (y-axis). **(C)** qRT-PCR of PSAT1 modified by Rylaze confirmed transcriptome analysis results. Results were normalized to corresponding control-treated cells and expressed as mean ± standard deviation (SD) (n=3). **(D)** MOLM-14 cells were treated with vehicle (DMSO), or Rylaze IC_50_ for 16 h. Cell lysates were prepared, followed by western blot analysis for PSAT1 expression. The bar diagram represents densitometric quantification of 3 independent experiments. **(E)** Approximately 18 h after plating, MOLM-14 and U937 AML cells were resuspended in vehicle (DMSO) or 0.1 µg/mL Rylaze for 72 h. After incubation, 10 µL of supernatant was added to a 96-well plate with the addition of the following reaction mixes to detect: Total Serine and Sample Background to the respective wells. Bar graphs represent the total serine concentration (µM) in the supernatant measured by fluorescence (n=3). ns, not significant; *=p<0.05, **=p<0.01, ***=p<0.001, ****=p<0.0001.

We performed Kyoto Encyclopedia of Genes and Genomes (KEGG) pathway and Gene Ontology (GO) term enrichment analysis to assess the similarities and differences in molecular processes associated with Rylaze treatment. The analysis provided information on the regulation of gene expression between vehicle control and Rylaze treatment in AML, as well as on the potential association with several putative pathways including serine family amino acids biosynthetic process (GO: 0009070, p-value = 2.1pE-08), amino acid transmembrane transport (GO:0003333, p-value = 5.10E-05) and regulation of programmed cell death (GO:0043067, p-value = 7.96E-06) (**Figure 1A**, **Supplementary Table S2**). Additionally, we assessed differences in transcriptional profiles between treatments to identify significant differentially expressed genes (DEGs), using a 5% false discovery rate and minimum 2-fold change cut-offs. We identified 114 significantly up-regulated and 27 significantly down-regulated genes following Rylaze treatment. Using a generalized linear model, we observed significant DEG changes following Rylaze (n = 141) monotherapy as compared to vehicle control, as shown in the heatmap derived from transcriptome data analysis [**Supplementary Figure S2D**, **Supplementary Table S3** (as a separate Excel database)]. The 141 DEGs identified in Rylaze treatment are illustrated in **Figure 1B** with up- and down-regulated genes highlighted in green (high) and pink (low), respectively.

We were intrigued when among those genes the levels of PSAT1, an important serine biosynthetic enzyme, were significantly increased in Rylaze-treated cells (p<0.001), supporting our hypothesis that Rylaze could induce *de novo* serine biosynthesis. This observed transcriptional change was further validated through quantitative PCR (qPCR) analysis (**Figure 1C**). To determine whether PSAT1 is also upregulated at the protein level, we conducted western blot analysis following a 16 h Rylaze treatment. As shown in **Figure 1D**, Rylaze treatment indeed significantly increased PSAT1 protein levels.

Furthermore, to validate the observation of increased serine following Rylaze treatment in AML, MOLM-14 and U937 cell lines were treated with vehicle (DMSO) or Rylaze for 72 h and the concentration of serine was measured by fluorescence (**Figure 1E**). We observed a dramatic increase in extracellular serine in the Rylaze treated cells for both cell lines, confirming that AML cells upregulate serine in response to Rylaze treatment.

### 3.3 Rylaze treatment upregulates serine biosynthesis through the amino acid response signaling pathway

**Figure 2:**
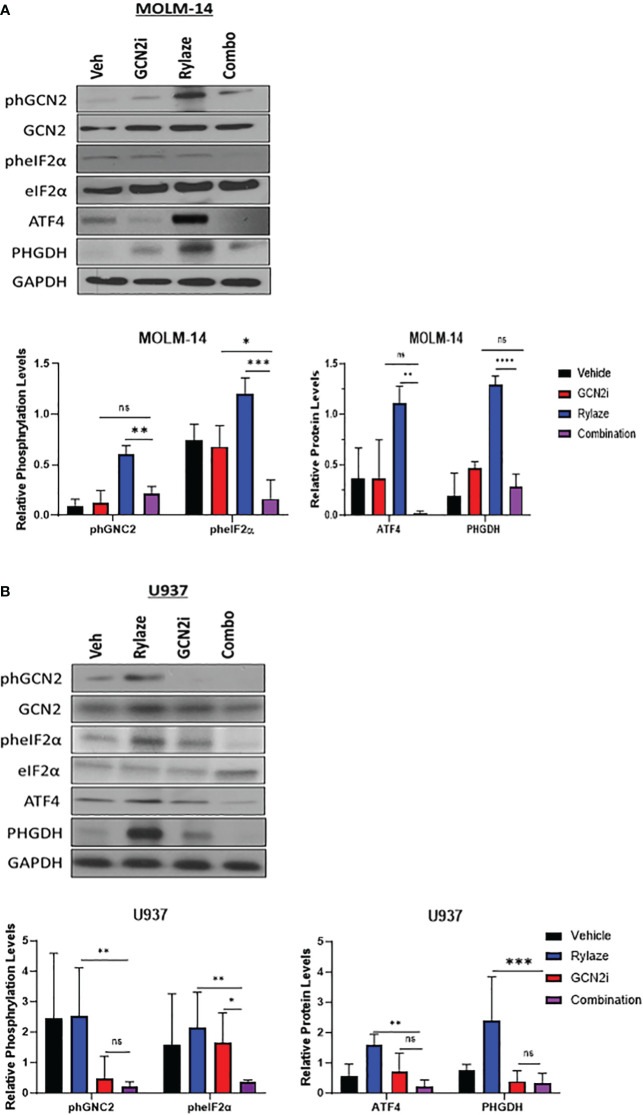
Rylaze promotes serine biosynthesis pathway. Representative immunoblots depicting the expression of amino acid response pathway proteins in MOLM-14 **(A)** and U937 **(B)** cell lines treated for 24 h with Rylaze (0.1 µg/mL), GCN2 inhibitor (2 µM), or the combination. The bar diagram represents densitometric quantification compared to vehicle control (n=3). ns, not significant, *=p<0.05, **=p<0.01, ***=p<0.001, ****=p<0.0001.

We next aimed to investigate the mechanism underlying the observed upregulation of serine biosynthesis following Rylaze treatment. One reasonable explanation is that Rylaze-induced nutrient restriction can trigger the initiation of the amino acid response (AAR) to maintain amino acid homeostasis. The AAR begins with the activation and autophosphorylation of General Control Nonderepressible 2 (GCN2), as it binds to accumulated uncharged tRNAs. GCN2 then phosphorylates eIF2α, thereby promoting and enhancing activating transcription factor 4 (ATF4) expression, leading to the upregulation of autophagy and amino acid biosynthesis. ATF4 is known to promote transcription of amino acid response elements, which include serine biosynthesis enzymes, PHGDH and PSAT1 (27–29). To determine if Rylaze-induced serine upregulation is mediated by the AAR pathway, we treated AML cell lines with Rylaze alone (0.1 µg/mL) or in combination with a small molecule inhibitor for GCN2 (GCN2-IN-1) and examined the effect on AAR pathway proteins and PHGDH expression, the enzyme that catalyzes the first step in the serine biosynthesis pathway. Rylaze treatment increased the phosphorylation of GCN2 and eIF2α and the expression of downstream ATF4 and PHGDH in both MOLM-14 and U937 cells (**Figures 2A, B**). Importantly, the combination of the GCN2 inhibitor with Rylaze resulted in a decrease in the Rylaze-mediated upregulation of PHGDH, suggesting that the Rylaze-induced upregulation of serine biosynthesis occurs through the activation of the AAR signaling pathway.

### 3.4 Silencing PHGDH inhibits AML cell proliferation and enhances the anti-AML effect of Rylaze

**Figure 3:**
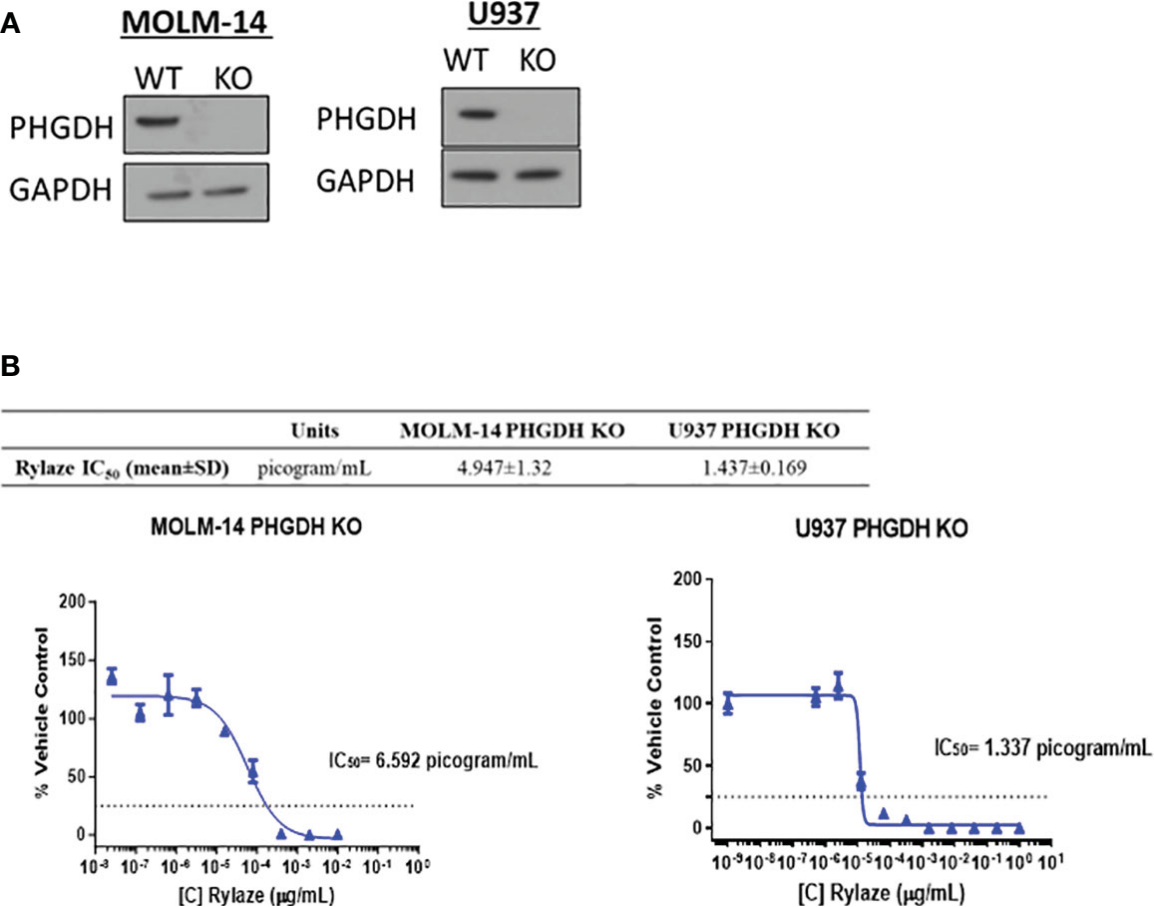
PHGDH silencing by CRISPR-*Cas9* enhances the anti-AML effects of Rylaze. **(A)** Representative immunoblots depicting expression of PHGDH in parental vs. PHGDH CRISPR-*Cas9* knockout cell lines (n=3). **(B)** Cells were exposed for 72 h to increasing doses of Rylaze and after WST-1 termination, IC_50_ values were calculated by GraphPad Prism. The table shows mean IC_50_ values (pg/mL) ± standard deviation (SD) from 3 independent experiments.

We next investigated the impact of interfering with serine metabolism in combination with Rylaze treatment. We focused on PHGDH since it is the rate-limiting enzyme in the *de novo* serine biosynthesis pathway, therefore controlling serine production (30). Furthermore, PHGDH has been shown to be amplified in breast cancer and multiple myeloma, further underscoring its suitability as an ideal target of serine biosynthesis (30, 31). Using CRISPR-*Cas9* technology, we knocked out PHGDH in MOLM-14 and U937 cells. The effect of knockout was validated by western blot analysis (**Figure 3A**). To assess whether interference with serine metabolism, potentiates the anti-AML activity of Rylaze, MOLM-14, and U937 PHGDH-knockout cell lines were treated with increasing doses of Rylaze (0-1 µg/mL). Remarkably, we observed a ~250-fold reduction (compare with data provided in the **Supplementary Figure S1A**) in the IC_50_ concentration, (MOLM-14: 4.9 ± 1.3 picogram (pg)/mL and U937: 1.4 ± 0.2 pg/mL) indicating a marked improvement in the anti-AML efficacy of Rylaze (**Figure 3B**). Collectively, these findings suggest that the inhibition of PHGDH effectively inhibits cell proliferation and amplifies the anti-AML effects observed with Rylaze treatment.

### 3.5 PHGDH inhibitor BI4916 synergizes with Rylaze to impede AML cell proliferation

**Figure 4:**
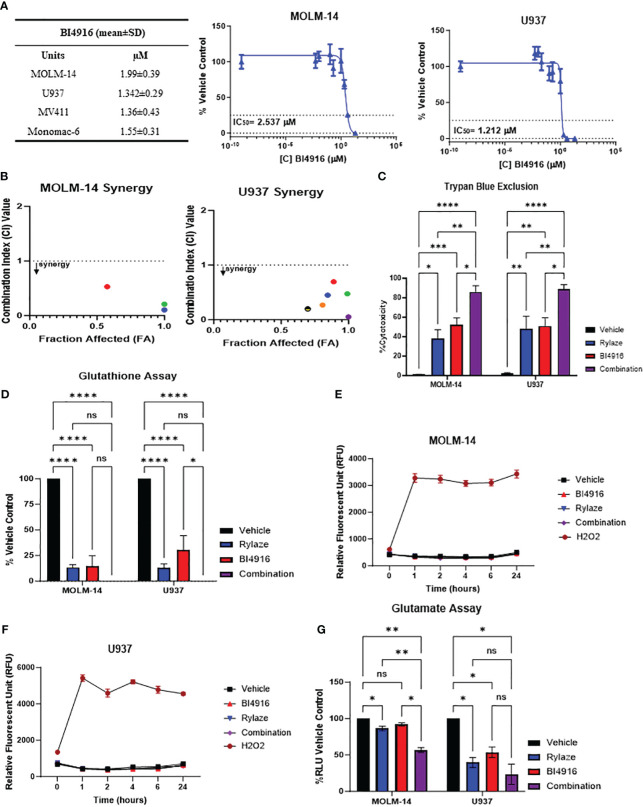
AML cells sensitive to PHGDH inhibition by small molecule inhibitor BI4916. **(A)** Approximately 18 h after plating, MOLM-14, U937, MV4-11 and MonoMac-6 cell lines were treated for 72 h with increasing doses of BI4916. Proliferation of cells was measured by the addition of WST-1. IC_50_s were generated by GraphPad Prism. The table shows BI4916 mean IC_50_ values (µM) ± standard deviation (SD) for the AML cell lines tested. **(B)** MOLM-14 and U937 cells were treated with fixed doses of BI4916 and Rylaze for 72 h. After WST-1 termination, combination indexes (CI) were calculated using Compusyn Software, using Chou Talalay’s method. CI values were calculated using Compusyn. **(C)** MOLM-14 and U937 cells were treated with BI4916 (2 µM), Rylaze (0.1 µg/mL), and BI4916-Rylaze treatment for 72 h and the percentage of cell death was measured using trypan blue exclusion, data is expressed as % cytotoxicity (n=3). **(D)** MOLM14 and U937 cells were treated as in **(C)** and GSH was measured by a luminescence-based assay 72 h post-treatment. Data is expressed as the percentage of GSH relative to vehicle control (n=3). MOLM-14 **(E)** and U937 **(F)** cells were pre-loaded with H_2_DCFA, a cell permeable indicator of ROS, then treated as in **(C)** or 200 µM of H_2_O_2_ as a positive control. ROS was measured at 0, 1, 2, 4, 6, and 24 h by plate reader. Results were normalized to control and expressed as mean ± standard error of the mean (SEM) (n=3). **(G)** MOLM-14 and U937 cell lines were treated as in **(C)** and glutamate levels were measured by a luminescence-based assay 72 h post treatment. Data is expressed as the percentage of Relative Light units (RLU) relative to vehicle control (n=3). ns, not significant, *=p<0.05, **=p<0.01, ***=p<0.001, ****=p<0.0001.

Having seen that silencing PHGDH enhances the anti-AML effect of Rylaze treatment, we next explored the pharmacological inhibition of PHGDH to interfere with serine biosynthesis. We chose to target PHDGH since inhibitors of PSAT1 are not commercially available. While several PHGDH inhibitors have been reported in the literature (25, 26), many of them lacked anti-proliferative activity in our AML model (data not shown), except for BI4916, a nicotinamide adenine dinucleotide (NADH/NAD^+^) competitive small molecule inhibitor of PHGDH (32). The dose curve response of BI4916 yielded the following IC_50_ concentrations (µM) in AML cell lines: MOLM-14 = 2 ± 0.4, U937 = 1.3 ± 0.3, MV4-11 = 1.4 ± 0.4, Monomac-6 = 1.6 ± 0.3 (**Figure 4A**), showing anti-AML effect. To determine if BI4916 and Rylaze synergize against AML cells, MOLM-14 and U937 cells were treated with serially-diluted BI4916 (0-50 µM) and Rylaze (0-1 µg/mL) alone and in fixed ratio combinations. Median effect analysis based on the Chou Talalay Method was used to generate combination index (CI) values, where a CI < 1 is synergistic, a CI = 1 is additive, and a CI > 1 is antagonistic (15). Confirming our hypothesis, the combinatory treatment of BI4916 and Rylaze synergistically inhibited AML cell growth at several of the tested combinations. For MOLM-14, the CI values for 3 of the combinations ranged between 0.1-0.52 and for U937, the CI values for 6 of the combinations ranged between 0.05-0.7 (**Figure 4B**).

We then evaluated the impact of this combined treatment approach on AML cell death. MOLM-14 and U937 cell lines were treated with BI4916 (2 µM), Rylaze (0.1 µg/mL), and their combination for 72 h and evaluated for cell death by trypan blue. As shown in **Figure 4C**, a significant increase in cell death in both cell lines was evident in the combination-treated group compared to each single-agent treatment.

### 3.6 BI4916 and Rylaze combination reduces glutathione levels

Glutathione (GSH), a major cellular antioxidant, is a tripeptide comprised of three amino acids: cysteine, glycine, and glutamate (13, 33). Given that glutamine is a precursor for glutamate, we wanted to determine if Rylaze-induced glutamine depletion would impact the production of cellular GSH levels. Furthermore, since serine biosynthesis functions as a precursor for the 1-carbon metabolic pathway, giving rise to both glycine and cysteine (13), we also wanted to determine if targeting PHGDH enhances the impact on GSH. MOLM-14 and U937 cells were treated with either BI4916, Rylaze, or the combination for 72 h and GSH levels were measured. We found that while both single agents significantly decreased the levels of GSH compared to vehicle control, the combination of BI4916 and Rylaze treatment further reduced GSH to undetectable levels (**Figure 4D**).

GSH undergoes conversion to its oxidative state (GSSG) while counteracting oxygen radicals. To investigate whether the observed decrease in GSH levels resulted from increased ROS, we assessed ROS levels using the H_2_DCFDA cell-permeable dye as a ROS indicator. Both MOLM-14 and U937 cell lines were exposed to either BI4916 (2 µM), Rylaze (0.1 µg/mL), or the combination, while hydrogen peroxide (H_2_O_2_) served as a positive control. Fluorescence readings were recorded at the indicated time points. Notably, our observations indicate that neither single-agent treatment nor the combination induced ROS production (**Figures 4E, F**), suggesting that the reduced GSH levels may be due to decreased glutamate, cysteine, and glycine production, rather than oxidative stress.

To determine whether intracellular glutamate levels are reduced in response to treatment, MOLM-14 and U937 cell lines were exposed to either BI4916 (2 µM), Rylaze (0.1 µg/mL), or the combination for 72 h and intracellular glutamate was measured. As expected, we found that Rylaze treatment significantly reduced glutamate levels in both cell lines (**Figure 4G**). The mechanism behind how inhibition of serine biosynthesis would impact intracellular glutamate is unclear at this time.

### 3.7 BI4916 and Rylaze synergize in primary human AML samples

**Figure 5:**
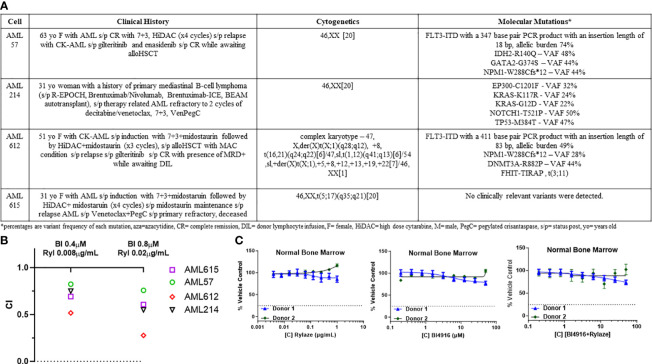
Combination of BI4916 and Rylaze demonstrates synergism and potent anti-proliferative effect on primary human AML samples. **(A)** Clinical course, cytogenetics and molecular mutations of patient-derived primary AML cells. **(B)** Primary AML cells were plated overnight and then treated with fixed ratios of BI4916 (called BI in the figure) and Rylaze (called Ryl in the figure) alone and in combination. Cultures were terminated at 48 h and viability was assessed with AlamarBlue. CI values were plotted with their respective combination ratios. **(C)** Healthy BMMC plated overnight then treated with increasing doses of BI4916, Rylaze or the combination. Cultures were terminated at 48 h and viability was assessed with Alamar Blue (n=2).

Building upon the observed combinatory effect between BI4916 and Rylaze in AML cell lines, we next explored their potential as a combinatory treatment in patient-derived primary AML cells with diverse mutational burdens (**Figure 5A**). After exposing primary AML cells to fixed ratios of BI4916 (0-50 µM) and Rylaze (0-1 µg/mL) for 48 h, viability was assessed using AlamarBlue and CI values were calculated using the Chou Talalay Method. CI values for the four primary AML patient samples ranged from 0.021 to 1 (**Supplementary Figures S3A-D**), among those doses two were reproduced in all four samples (**Figure 5B**), indicating a synergistic inhibition of AML cell growth with the combination of BI4916 and Rylaze. Notably, when we treated patient-derived healthy bone marrow mononuclear cells (BMMC), neither the single-agent nor combination treatment of BI4916 and Rylaze induced any toxic effects (**Figure 5C**), pre-clinically suggesting a potential clinical selectivity of this therapeutic approach.

### 3.8 BI4916 and Rylaze combination decreases protein synthesis and cap-dependent mRNA translation

**Figure 6:**
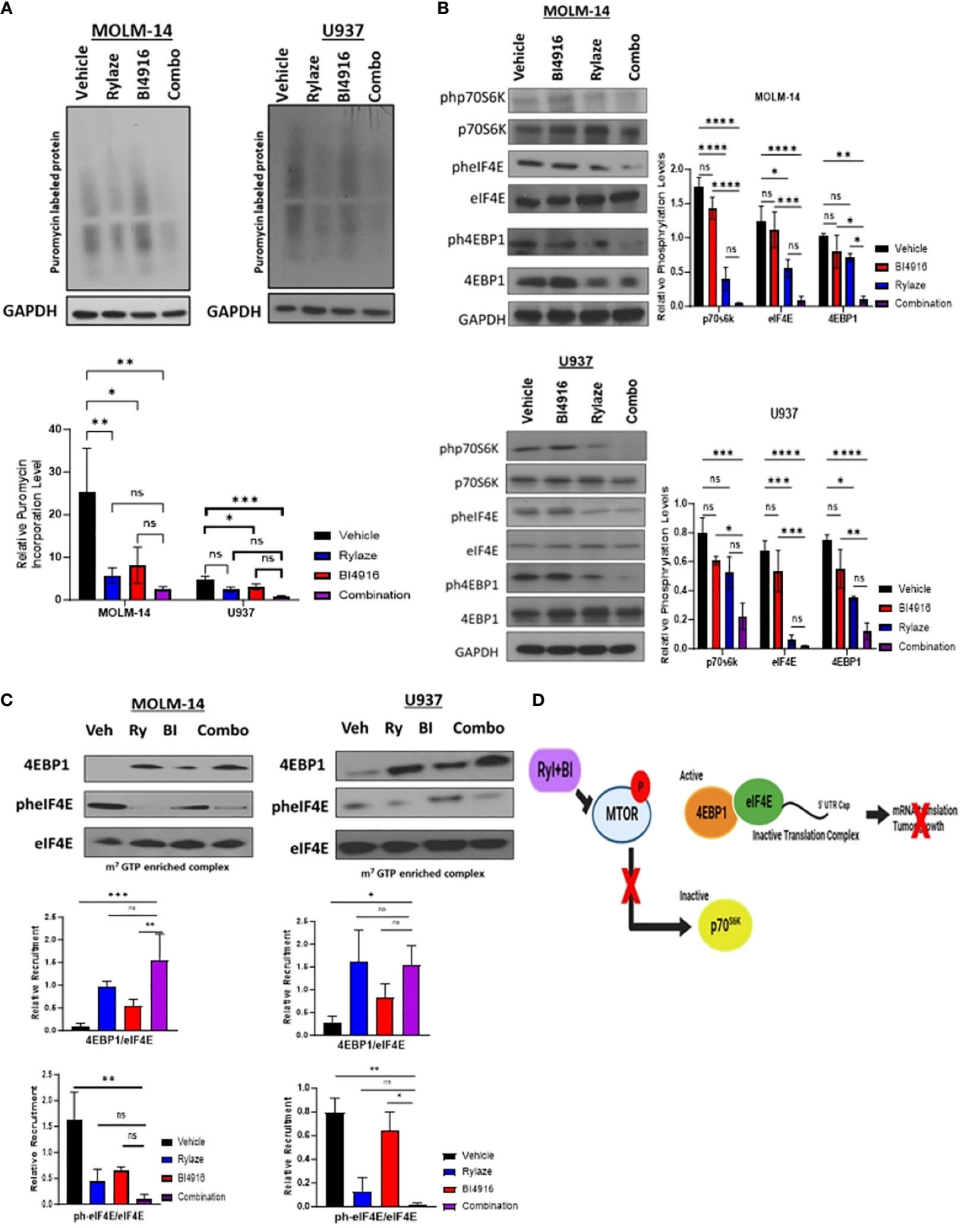
BI4916 and Rylaze impedes cap-dependent translation and protein synthesis. **(A)** MOLM-14 and U937 were treated with BI4916 (2 µM), Rylaze (0.1 µg/mL) and BI4916-Rylaze for 16 h followed by 20 min of incubation with puromycin (1 µg/mL) and then lysed. Cell lysates were subjected to immunoblotting with the anti-puromycin antibody (SUnSET [surface sensing of translation] assay). The bar diagram represents densitometric quantification of 3 independent experiments. **(B)** MOLM-14 and U937 cells were treated as in **(A)**, and cellular lysates were probed with the indicated antibodies. Bar diagram represents densitometric quantification of experiments (n=3). **(C)** Cellular lysates of MOLM-14 and U937 treated as in **(A)** were incubated with m^7^GTP sepharose beads for 2 h, followed by immunoblotting and probing with the indicated antibodies. The bar diagram represents densitometric quantification (n = 3). **(D)** Schematic of proposed mechanism of BI4916-Rylaze treatment on cap-dependent mRNA translation made with BioRender. ns, not significant, *=p<0.05, **=p<0.01, ***=p<0.001, ****=p<0.0001.

Prior reports by our group have demonstrated that crisantaspase treatment diminishes protein synthesis in AML by interfering with cap-dependent mRNA translation downstream of mammalian target of rapamycin (mTOR) signaling, specifically by inhibiting the phosphorylation of p70 ribosomal S6 kinase (p70S6K), a key downstream effector of mTOR (6, 7). A critical aspect of cap-dependent translation initiation involves the presence of eIF4E, which participates in the eIF4F initiation complex by binding to 5´ mRNA caps (34). Upon activation of the mTOR signaling pathway, the eIF4E repressor protein, 4EBP1, undergoes phosphorylation and deactivation, allowing eIF4E to engage in the cap-binding complex and promote translation initiation. Conversely, inhibition of the mTOR signaling pathway results in the binding of unphosphorylated 4EBP1 to eIF4E, leading to the prevention of cap-binding complex formation and translation (35). To understand the mechanistic implications of the BI4916 and Rylaze combination on mTOR signaling and protein synthesis, we first assessed global protein synthesis using puromycin incorporation. MOLM-14 and U937 cells were treated with BI4916 (2 µM), Rylaze (0.1 µg/mL), and their combination for 16 h, followed by a short exposure to puromycin. Whole-cell lysates were probed with an anti-puromycin antibody to detect puromycin incorporation into newly synthesized polypeptide chains. The combinatory treatment with BI4916 and Rylaze resulted in a significant reduction in overall protein synthesis in the combination group (**Figure 6A**). It is important to note that the vehicle group in our experiments acts as control, allowing us to discern the impact of Rylaze and combination treatments on protein synthesis by contrasting them with untreated cells (**Supplementary Figure S4**).

**Figure 7:**
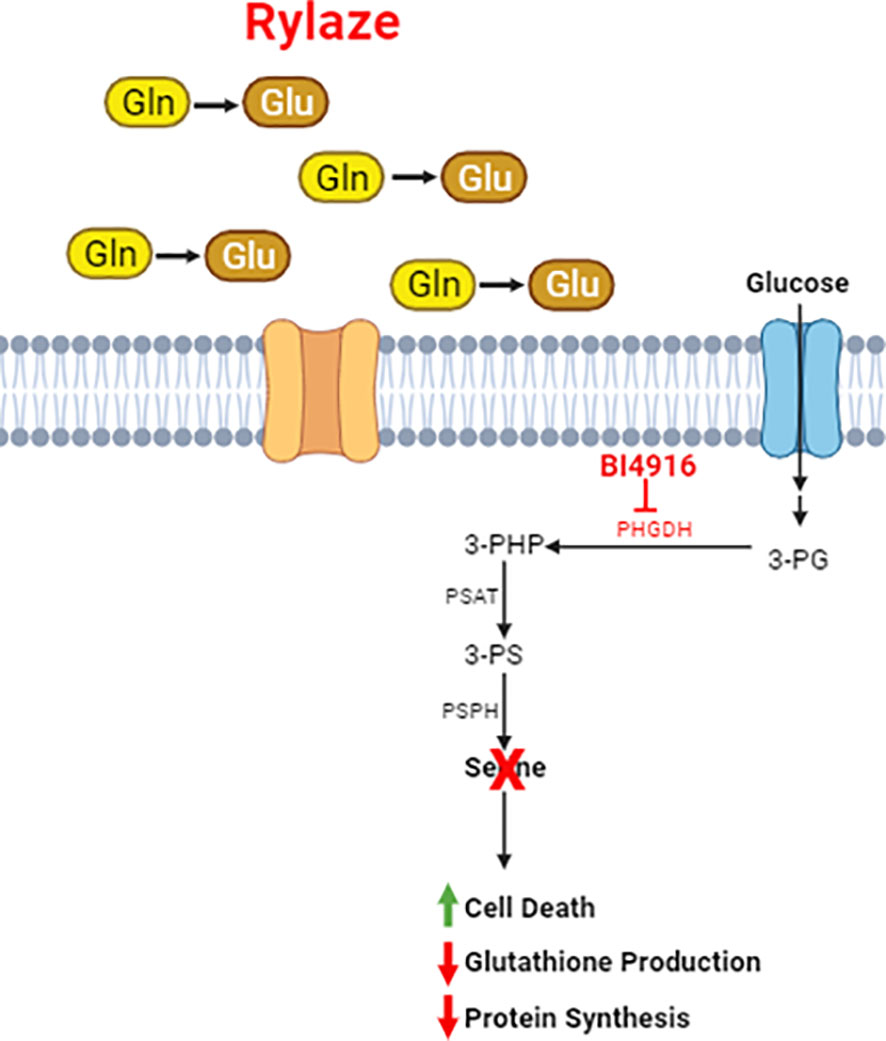
Schematic of Rylaze and BI4916 effect on AML cells. Phosphoglycerate Dehydrogenase (PHGDH) regulates *de novo* serine biosynthesis using glycolytic intermediate 3-phosphoglycerate (3-PG). An oxidative reaction converts 3-PG to 3-phosphohydroxypyruvate which leads to *de novo* serine biosynthesis. Targeting PHGDH with BI4916 inhibits *de novo* serine biosynthesis which synergizes with glutamine deprivation mediated by Rylaze (crisantaspase). BI4916-Rylaze combination leads to decreased glutathione production and protein synthesis and increased cell death.

To investigate whether this decrease in protein synthesis is linked to the inhibition of cap-dependent mRNA translation downstream of mTOR signaling, we examined the phosphorylation status of the key mTOR signaling pathway proteins in response to BI4916-Rylaze combinatory treatment. A significant reduction in the phosphorylation of p70S6K, 4EBP1, and eIF4E following combinatory treatment was found (**Figure 6B**). Subsequently, to assess recruitment levels of 4EBP1 and eIF4E on the cap-binding complex, we conducted m^7^GTP enrichment experiments. In our combinatory treatment groups, we observed increased recruitment of 4EBP1 to the cap complexes and a significant reduction in eIF4E phosphorylation (**Figure 6C**) compared to vehicle control, indicating inactivation of translation initiation complex (see proposed mechanism **Figure 6D**). Collectively, our findings demonstrate that Rylaze-mediated glutamine depletion synergizes with the serine biosynthesis inhibitor BI4916, resulting in the inhibition of AML cell growth and glutathione production, as well as a reduction in protein synthesis, particularly through the inhibition of cap-dependent mRNA translation (**Figure 7**).

## 4 Discussion

Our previous work has identified the reproducible upregulation of serine in the plasma following crisantaspase treatment in both mouse and human AML samples (5). Expanding on our previous findings, here we report that crisantaspase treatment induces activation of the AAR signaling pathway, resulting in the upregulation of serine biosynthesis enzymes. Furthermore, we found that targeting serine biosynthesis in combination with crisantaspase treatment synergistically inhibits leukemia cell growth in AML cell lines and primary patient samples.

Notably, the major sources of serine in human are i) dietary intake, ii) degradation of endogenous proteins, iii) *de novo* synthesis from 3-phosphoglycerate and iv) glycine (36). Approximately 75% of the plasma serine in fasting humans is generating from 3-phosphoglycerate and glycine (37). *De novo* serine biosynthesis involves a three-step enzymatic process that initiates with the NAD^+^-dependent oxidation of the glycolytic intermediate 3-phosphoglycerate (3-PG) into 3-phosphohydroxypyruvate (3-PHP). This initial step is facilitated by PHGDH. 3-PHP then is transformed into 3-phosphoserine (3-PS) through a glutamate-dependent transamination reaction catalyzed by phosphoserine aminotransferase 1 (PSAT1). Lastly, phosphoserine phosphatase induces the hydrolysis of 3-PS, leading to the production of serine (14).

3-PG is synthesized during glycolysis or gluconeogenesis (37) and 3-PS is synthesized through a transamination process (catalyzed by PSAT) with glutamate. This process is the source of nitrogen, but not carbon, in serine from glutamate/glutamine.

Serine metabolism has emerged as a crucial participant in cancer biology, and PHGDH, the rate-limiting enzyme of *de novo* serine biosynthesis has garnered attention as a therapeutic target (12, 14, 38). Elevated levels of PHGDH expression have been observed in various solid tumors, including triple-negative breast cancer, melanoma, and lung adenocarcinoma, and inhibiting this enzyme has demonstrated the ability to impede cancer cell proliferation (39–43). Our observed upregulation of the serine biosynthesis pathway in response to glutamine withdrawal through the AAR sheds light on a potentially important mechanism in AML cells’ adaptation to nutrient deprivation. The AAR is a well-known cellular stress response mechanism that allows cells to maintain essential amino acid levels during nutrient limitations by activating various metabolic pathways (29, 44). This adaptive response, triggered by Rylaze treatment, potentially enables AML cells to maintain essential biosynthetic pathways, achieve amino acid homeostasis, and escape cell death. Targeting this adaptive response to improve Rylaze efficacy is a novel therapeutic strategy that has the potential to impact not only AML treatment approaches, but any other cancer model in which glutamine-targeting strategies are being investigated. Notably, the work by Polet et al., showcases a similar theme of interconnected amino acid metabolism manipulation for enhanced therapeutic outcomes. In their study, the authors demonstrate that diminishing serine availability in combination with L-asparaginase synergistically hinders leukemia cell proliferation (12).

Dysregulated translation is a hallmark of cancer cells, enabling them to sustain uncontrolled growth and proliferation (45). Recent studies suggested the therapeutic potential of targeting translation as a strategy to combat some cancers (35). Previous research from our group has demonstrated the downregulation of the mTOR pathway and the resulting inhibition of cap-dependent translation in AML cells upon treatment with a long-acting crisantaspase formulation (7). The mTOR pathway plays a pivotal role in regulating cellular translation, and its dysregulation is frequently observed in cancer, including AML (46). Our findings not only corroborate the significance of targeting translation in cancer therapy but also expand upon this narrative. By simultaneously targeting serine and glutamine metabolism, we disrupt the amino acid balance crucial for efficient protein synthesis (35), and observe a substantial reduction in mRNA translation and protein synthesis. This approach enhances the perturbation of translation machinery and further suppresses the ability of AML cells to sustain their uncontrolled growth and proliferation.

Our transcriptomic analysis revealed significant alterations in several pathways following the treatment of Rylaze in AML. Although the upregulation of PSAT1 was the focus of this study, Rylaze treatment upregulated other notable genes including CHAC1, ADM2, CTH, and S100P. Among these, CHAC1, an ATF4 target gene activated by the amino acid response pathway, is involved in glutathione degradation and ferroptosis (47, 48). The upregulation of CHAC1 following PHGDH silencing has been implicated in breast cancer, and in AML; its overexpression is associated with high-risk prognosis and reduced overall survival (47, 49). Conversely, S100P, a calcium-binding protein, showed positive correlation with overall survival in AML patients, suggesting its potential as a novel biomarker and prognostic factor in AML (50). Ongoing work in our lab is focused on investigating role of these genes after Rylaze treatment and their potential application to future AML therapeutic strategies.

Collectively, these findings contribute to the expanding body of knowledge about metabolic alterations in cancer and further support the anti-AML activity of crisantaspase. Furthermore, it underscores the potential of targeting serine metabolism as a promising therapeutic strategy in AML patients, especially when paired with the FDA-approved crisantaspase. In light of these findings, and evidence that BI4916 has intrinsic instability *in vivo* (32), ongoing effort is also focused on the development of a novel and potent PHGDH inhibitor for testing in *in vivo* AML models.

## Data availability statement

The datasets presented in this study can be found in online repositories. The names of the repository/repositories and accession number(s) can be found in the article/**Supplementary Material**.

## Ethics statement

The studies involving humans were approved by University of Maryland Baltimore Institutional Review Board. The studies were conducted in accordance with the local legislation and institutional requirements. The participants provided their written informed consent to participate in this study. Ethical approval was not required for the studies on animals in accordance with the local legislation and institutional requirements because only commercially available established cell lines were used.

## Author contributions

KH: Conceptualization, Data curation, Formal analysis, Investigation, Methodology, Software, Validation, Writing – original draft, Writing – review & editing. DB: Writing – review & editing, Writing – original draft. AS: Data curation, Formal analysis, Software, Writing – review & editing. BC-C: Methodology, Writing – review & editing. RL: Writing – review & editing. AE: Conceptualization, Funding acquisition, Project administration, Resources, Supervision, Writing – review & editing, Writing – original draft.
