# Supplementary material for: Dual targeting of glutamine and serine metabolism in acute myeloid leukemia
Source: Front Oncol. 2024 Apr 16;14:1326754. doi: 10.3389/fonc.2024.1326754 (PMC11059989; doi:10.3389/fonc.2024.1326754)
Supplement: Supplementary file 1 [file DataSheet_1.pdf]

Supplementary Material

1 Supplementary Figures and Tables

1.1 **Supplementary Fig. S1.** Rylaze inhibits AML cell growth and proliferation.

1.2 **Supplementary Fig. S2.** Alignment statistics and heatmap for transcriptome sample.

1.3 **Supplementary Fig. S3.** Synergistic Effects of BI4916 and Rylaze Treatment in AML Patient Samples

1.4 **Supplementary Fig. S4.** Cell viability of AML cells post BI4916-Rylaze combination treatment

1.5 **Supplementary Table S1.** Transcriptome alignment summary

1.6 **Supplementary Table S2.** Gene enrichment analysis summary

1.7 **Supplementary Table S3.** Transcriptome data analysis [Separate Excel File]

1.1 Supplementary Figure S1.

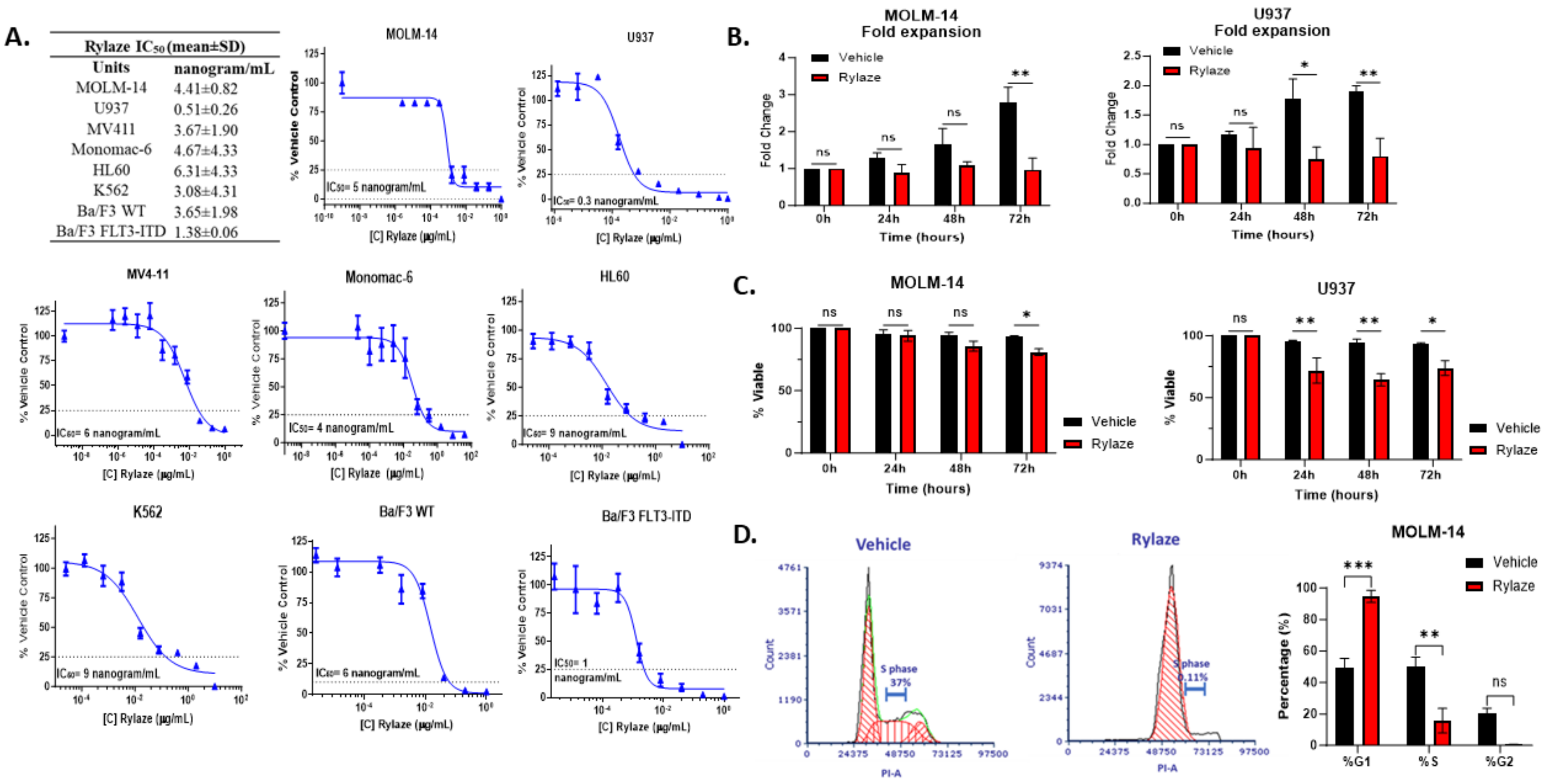

E.

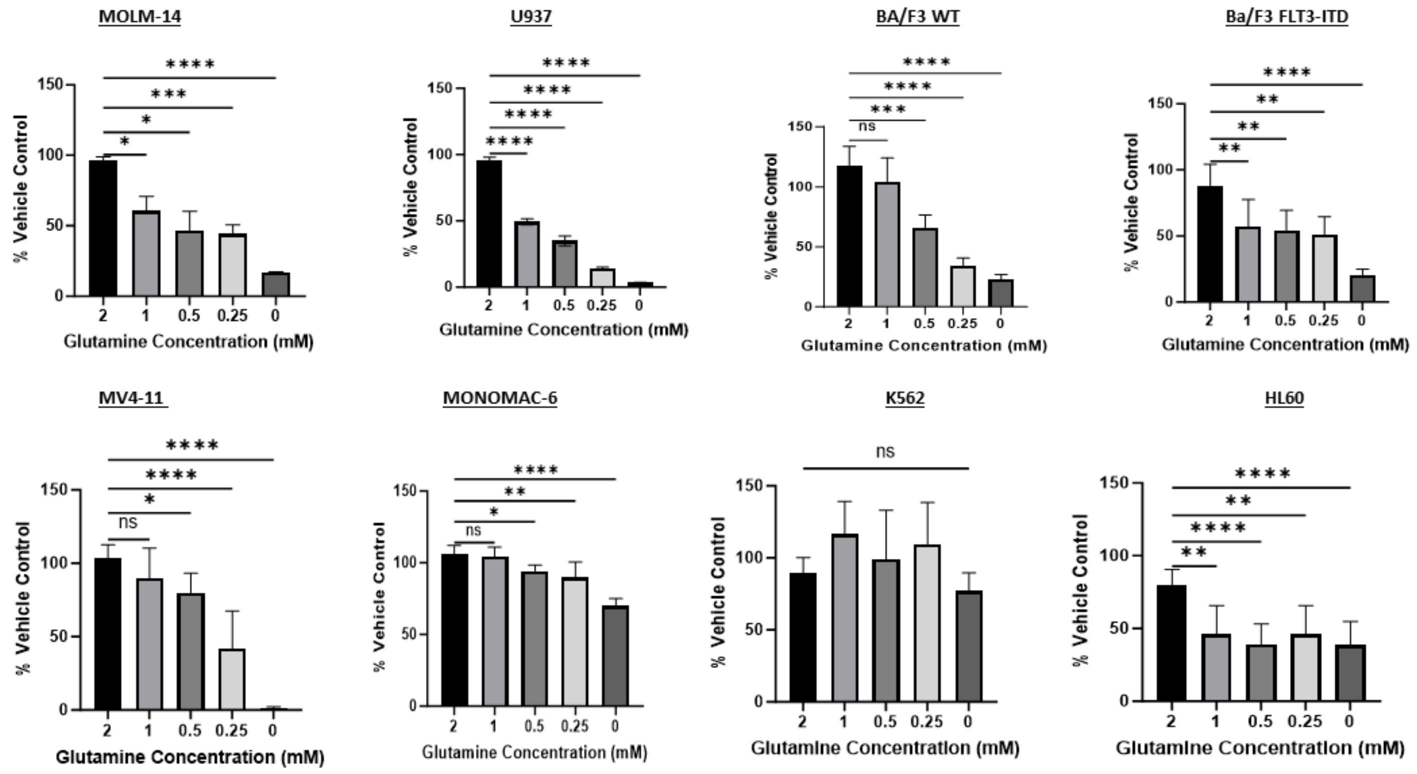

Supplementary Figure S1. Rylaze inhibits AML cell growth and proliferation. (A) Approximately 18 h after plating, human AML cell lines were treated for 72 h with Rylaze. Proliferation of cells was measured by the addition of WST-1. IC$_{50}$s were generated by GraphPad Prism (n=3). The table shows Rylaze IC$_{50}$ values ± standard deviation (SD) for all 8 AML cell lines tested. (**B**) Bar graphs represent the fold change in expansion of the cells incubated for 24, 48, and 72 h in media with and without Rylaze (0.1 µg/mL), measured by trypan blue exclusion (n=3). **(C)** Bar graphs represent the % viability of cells treated for 24, 48, and 72 h with vehicle (DMSO) or Rylaze (0.1 µg/mL), measured by trypan blue exclusion. **(D)** MOLM-14 cells were treated with vehicle (DMSO) and Rylaze (IC$_{50}$) for 48 h. Cells were fixed and stained with propidium iodide (PI), then analyzed by flow cytometry (n=3). **(E)** Approximately 18h after plating, leukemia cell lines were cultured in media with decreasing doses of glutamine (mM) for 72 hours. Proliferation of cells was measured by addition of WST-1. Bar graphs represent the percent of cell growth compared to control cells cultured in normal growth media. ns= not significant, *=p<0.05, **=p<0.01, ***=p<0.001

1.2 Supplementary Figure S2.

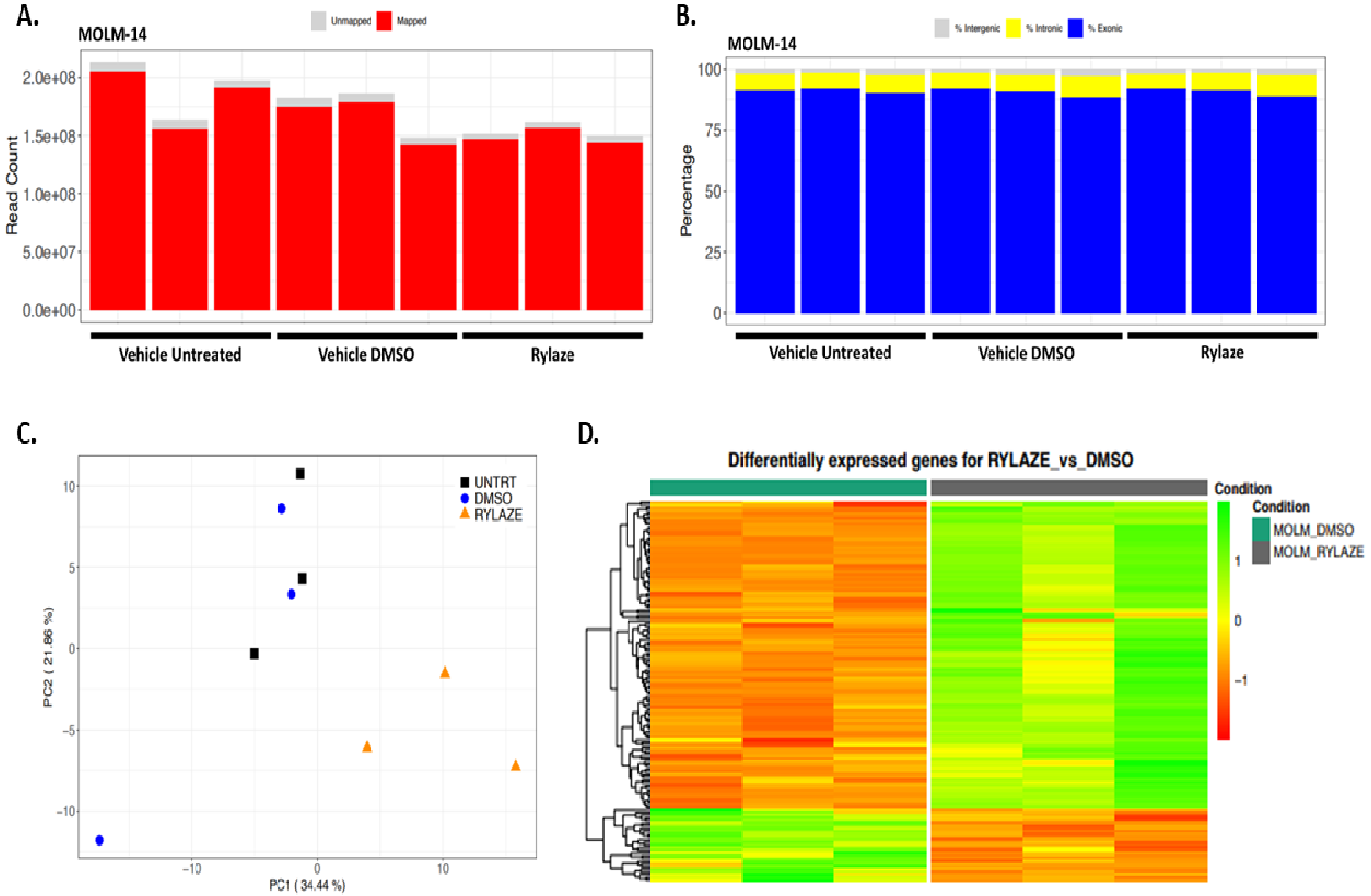

Supplementary Figure S2. Alignment statistics and heatmap for transcriptome samples. (A) The bar plot summarizes the alignment statistics for the transcriptome dataset. The number of reads that mapped (red) and did not map (grey) to the human reference genome for each sample. **(B)** The proportion of mapped reads that aligned to different regions in the genome, including exonic (blue), intronic (yellow), and intergenic (grey) regions. **(C)** Principal component analysis (PCA) based on gene expression in control and treatment samples using the MOLM-14 cell line**. (D)** Heatmap illustrating the gene expression of the DEGs identified between Rylaze treatment and control DMSO. High expression and low expression can be identified using a green-to-red gradient scale. UNTRT=untreated.

1.3 Supplementary Figure S3.

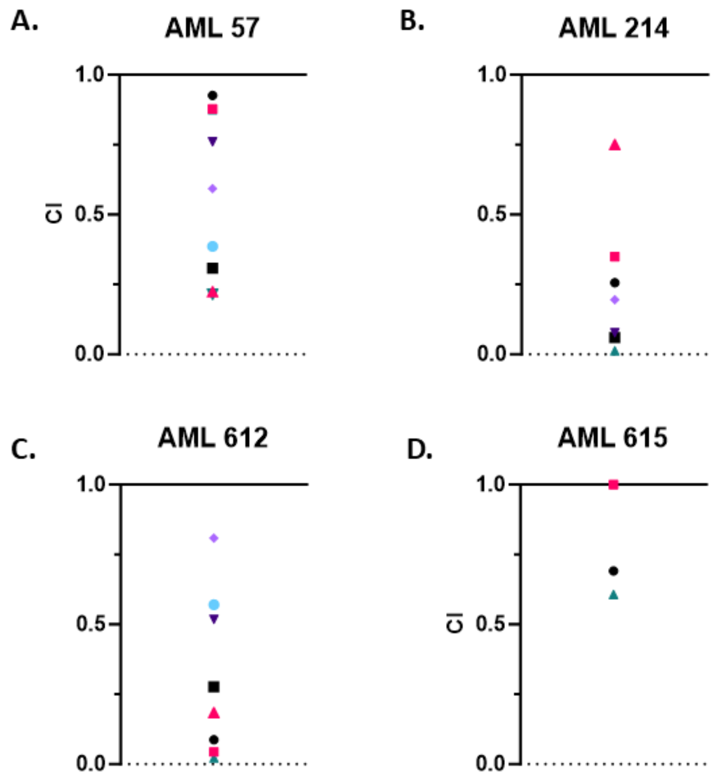

Supplementary Figure S3. Synergistic Effects of BI4916 and Rylaze Treatment in AML Patient Samples. (A-D) Primary AML cells were plated overnight and then treated with fixed ratios of BI4916 and Rylaze alone and in combination. Cultures were terminated at 48 h and their viability was assessed with AlamarBlue. Combination Index (CI) were plotted for each patient sample. Each symbol represents a combination ratio of BI4916 and Rylaze.

1.4 Supplementary Figure S4.

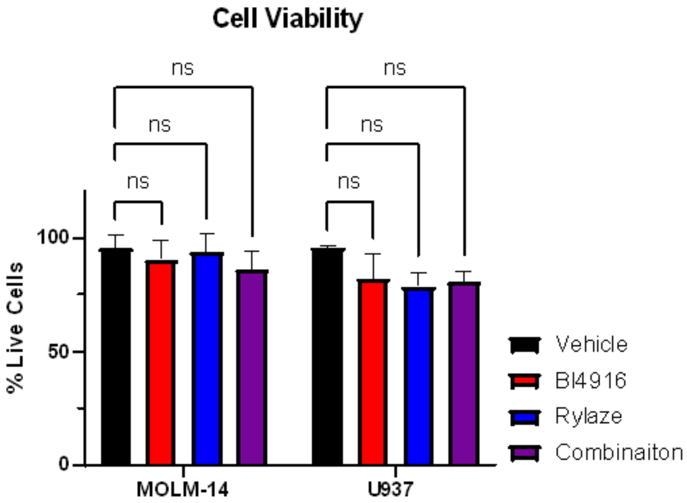

Supplementary Figure S4. Cell viability of AML cells post BI4916-Rylaze combination treatment. Bar graphs represent the % viability of cells treated for 16 hours with vehicle (DMSO), BI4916 (2µM), Rylaze (0.1µg/mL), and their combination, measured by trypan blue exclusion.

1.5 Supplementary Table S1.

Supplementary Table S1. Transcriptome alignment summary								
**#Sample_ID**	**Total Reads**	**Total Mapped Reads**	**Percent Mapped Reads**	**Percent Properly Paired**	**Uniquely Mapped Reads**	**Percent Exonic**	**Percent Intronic**	**Percent Intergenic**
**Vehicle_1**	213225790	205082523	95.17	96.33	282548969	91.13	7.03	1.84
**Vehicle_2**	162858150	155956593	95.70	96.30	191741286	91.97	6.41	1.62
**Vehicle_3**	197386560	191404564	95.99	96.43	170431507	90.07	7.77	2.16
**VehicleD_1**	181931432	175119538	96.26	96.45	159012394	91.91	6.34	1.75
**VehicleD_2**	186371844	179046713	96.07	96.36	158559855	91	6.81	2.19
**VehicleD_3**	147979856	142658457	96.41	96.6	124891303	88.49	8.77	2.74
**Rylaze_1**	151569652	147039859	97.01	96.45	132231321	91.94	6.16	1.90
**Rylaze_2**	161663164	156612900	96.88	96.45	141958178	91.31	6.95	1.74
**Rylaze_3**	149663164	144067703	96.21	96.38	129012435	88.90	8.69	2.41

1.6 Supplementary Table S2.

Supplementary Table S2. Gene Enrichment Summary								
**ID**	**Description**	**ONTOLOGY**	**GeneRatio**	**BgRatio**	**Count**	**pvalue**	**p.adjust**	**geneID**
GO:0009070	serine family amino acid biosynthetic process	BP	5/96	19/20610	5	2.18E-08	7.01E-05	CTH/PSAT1/PSPH/ CBS/CBSL
GO:0019343	cysteine biosynthetic process via cystathionine	BP	3/96	3/20610	3	9.79E-08	0.0001575	CTH/CBS/CBSL
GO:0009069	serine family amino acid metabolic process	BP	6/96	52/20610	6	1.49E-07	0.0001601	CTH/PSAT1/PSPH/ CBS/SLC7A11/CBSL
GO:0008652	cellular amino acid biosynthetic process	BP	7/96	90/20610	7	2.08E-07	0.0001670	CTH/PSAT1/ASNS/ PSPH/GPT2/CBS/CBSL
GO:0006563	L-serine metabolic process	BP	4/96	13/20610	4	3.06E-07	0.0001968	PSAT1/PSPH/CBS/CBSL
GO:0009092	homoserine metabolic process	BP	3/96	5/20610	3	9.73E-07	0.0003497	CTH/CBS/CBSL
GO:0019344	cysteine biosynthetic process	BP	3/96	5/20610	3	9.73E-07	0.0003497	CTH/CBS/CBSL
GO:0019346	transsulfuration	BP	3/96	5/20610	3	9.73E-07	0.0003497	CTH/CBS/CBSL

1.7 Supplementary Table S3. Transcriptome data analysis [Separate Excel file]. Differentially expressed genes identified across multiple comparisons between Vehicle, and Rylaze -treatment with their corresponding information about their log2-transformed fold-change (LFC), FDR-adjusted p-value (FDR), and differential expression status (DE, 0 = non-DE, 1 = DE) across all comparisons.
